# Bayesian ridge regression shows the best fit for SSR markers in *Psidium guajava* among Bayesian models

**DOI:** 10.1038/s41598-021-93120-z

**Published:** 2021-07-01

**Authors:** Flavia Alves da Silva, Alexandre Pio Viana, Caio Cezar Guedes Correa, Eileen Azevedo Santos, Julie Anne Vieira Salgado de Oliveira, José Daniel Gomes Andrade, Rodrigo Moreira Ribeiro, Leonardo Siqueira Glória

**Affiliations:** 1 Laboratory of Plant Genetic Breeding (LMGV), Center for Agricultural Sciences and Technologies (CCTA), Universidade Estadual do Norte Fluminense Darcy Ribeiro (UENF), Av. Alberto Lamego 2000, Campos dos Goytacazes, Rio de Janeiro, 28013-602 Brazil; 2 Laboratory of Animal Science (LZO), Center for Agricultural Sciences and Technologies (CCTA), Universidade Estadual do Norte Fluminense Darcy Ribeiro (UENF), Av. Alberto Lamego 2000, Campos dos Goytacazes, Rio de Janeiro, 28013-602 Brazil

**Keywords:** Computational biology and bioinformatics, Plant sciences

## Abstract

Markers are an important tool in plant breeding, which can improve conventional phenotypic breeding, generating more accurate information outcoming better decision making. This study aimed to apply and compare the fit of different Bayesian models BRR, BayesA, BayesB, BayesB (setting the value from very low to $\pi$ = ${10}^{-5}$), BayesC and Bayesian Lasso (LASSO) for predictions of the genomic genetic values of productivity and quality traits of a guava population. The models were fitted for traits fruit mass, pulp mass, soluble solids content, fruit number, and production per plant in the genomic prediction with SSR markers, obtained through the CTAB extraction method with 200 primers. The Bayesian ridge regression model showed the best results for all traits and was chosen to predict the individual’s genomic values according to the cross-validation data. A good stabilization of the Markov and Monte Carlo chains was observed with the mean values close to the observed phenotypic means. Heritabilities showed good predictive accuracy. The model showed strong correlations between some traits, allowing indirect selection.

## Introduction

Tropical fruits have a great commercial value worldwide because, besides being widely consumed in the countries that produce them, they are highly appreciated and with a great added value around the world^1^. One of these perennial fruits is the guava tree (*Psidium guajava* L.) which is gaining space on the market in parts, due to the increasingly efficient selection methods for improving the species. One of these methods is the selection of superior individuals embased by molecular markers, such as genomic selection. This method characterizes the ideal association between conventional breeding based on phenotypic observations and modern molecular techniques currently available. Its use has a great impact on breeding programs allowing better planning by obtain more accurate and precise estimates^2^.

However, the breeder has available several statistical models to associate the marks with the phenotypes, which makes it a challenge to choose a suitable model for the response of the species and marks. Recently, among these models, Bayesian approaches have gained a lot of prominence with the advent of computational power. With a Bayesian approach, the effects of the markers can be estimated together to predict the genomic values for a quantitative trait without making the previous selection in the panel of markers^3^. This Bayesian genomic selection has as main advantages the inclusion of a priori information in the model, besides generating more accurate credibility intervals^4^.

The accuracy varies between models of genomic selection, according to their assumptions and treatments of the effects of the markers. For example, it was identified that Bayesian models (*Bayesian LASSO—BL*) and ridge regression models (BRR) showed superior performance for traits controlled by additive genetic effects^5^.

Among the available Bayesian approaches, we can mention LASSO Bayesian—BL that combines both selection and trait contraction methods. Advantageous concerning the most common method that does not use trait selection. It has an exponential priori in the variance of the markers, resulting in a double exponential distribution. The double exponential distribution has a high mass density at zero, and heavier priori tails compared to a Gaussian distribution^6,7^. Bayesian ridge regression—BRR induces homogeneous shrinkage of all marker effects to zero and produces a Gaussian distribution of marker effects^8^.

Another model is BayesA, that uses an inverse-chi-square (× 2) in the variance of the markers, producing a scaled t distribution for the effects of the markers. Similar to BL and unlike BRR, it shrinks the markers with small effects to values close to zero, and the markers with greater effects are maintained. The final distribution of the marks shows a higher peak of mass density close to zero compared to the double exponential distribution^6,9^. BayesB is similar and uses an inverse x^2^ but uses shrinkage and selection methods of the trait. And when the priori parameter π = 0, it is like BayesA^10^. BayesC also applies the shrinkage and selection methods of trait and generates a Gaussian distribution of the effects of the markers. BayesB and BayesC consist of close to zero density in the distribution when using low priori^11^.

For the breeder, finding out which model best fits his object of study is of paramount importance for the planning of the breeding program. For guava, there is not yet a study looking for which model is best applied to the association of marks, although primers for simple-sequence repeats (SSR) have also been applied, as observed in Dinesh, et al.^12^.

This study aimed to apply and compare the fit of different Bayesian models BRR, BayesA, BayesB, BayesB (setting the value from very low to $\pi$ = ${10}^{-5}$) and BayesC and Bayesian Lasso (LASSO) for predictions of the genomic genetic values of productivity and quality traits of a guava population.

## Material and methods

### Genetic material

The data used in this study were obtained in the experiment carried out from Guava Breeding Program at State University of Northern Rio de Janeiro, in accordance with the institutional guidelines for carrying out experiments. The experimental area was located at the Antônio Sarlo Technical and Agricultural School, in Campos dos Goytacazes, Rio de Janeiro, Brazil, situated at 21° 08′ 02″ S and 41° 40′ 47″ W, with a sub-humid and dry tropical climate, with an average temperature between 22 and 25 °C, and an average annual precipitation of 1200 mm. In the experimental field, a complete block design with two replications was used. Each plot contained one of the seventeen guava segregating families with twelve plants (full siblings).

The families were obtained by crossings between some accessions, that were established considering information on genetic diversity obtained by Pessanha et al.^13^. Were selected the best plants from each family based on the work of Silva et al.^14^ to apply the markers, were:

UENF 1834 × UENF 1833 (12 plants); UENF 1831 × UENF 1830 (12 plants); UENF 1831 × UENF 1832 (1 plant); UENF 1833 × UENF 1832 (11 plants); UENF 1834 × UENF 1839 (1 plant); UENF 1835 × UENF 1834 (16 plants); UENF 1836 × UENF 1835 (15 plants); UENF 1833 × UENF 1836 (2 plants); UENF 1831 × UENF 1835 (10 plants); UENF 1833 × UENF 1835 (5 plants); UENF 1834 × UENF 1837 (5 plants); UENF 1832 × UENF 1835 (6 plants).

These plants were selected for their performance on seven years of harvests, and represent the plants who will proceed to the next stages of the breeding program. In each plant, some traits were measured (n = 5): fruit mass in g (FM), pulp mass in g (PM), soluble solids content in °Bx (SSC), number of fruits per plant (NF), and production per plant (PROD). In the same plants, were collected young leaves for DNA extraction.

### DNA extraction and quantification

DNA extraction was carried out using the standard CTAB method with modifications^15^. Then, the DNA was quantified by analysis on 1% agarose gel on TAE 1X buffer (Tris, Sodium Acetate, EDTA, pH 8.0), using the Lambda marker (λ) of 100 bp (100 ng μL^−1^) (Invitrogen, USA), by comparing the bands. For this procedure, the samples were stained using the mixture of Gel, RedTM, and Blue Juice (1:1), and the image was captured by the MiniBis Pro photocumentation system (Bio-Imaging Systems). Subsequently, the DNA samples were diluted to a working concentration of 10 ng μL^−1^.

### Polymerase chain reaction (PCR)

The PCR reactions were carried out in thermocyclers from Applied Biosystems/Veriti 96 well, in a 38 cycle program, obeying the following temperatures and time: 94 °C for one minute (initial denaturation), 94 °C for two minutes (cyclic denaturation), the specific temperature of each initiator, in °C, for one minute (annealing), 72 °C for three minutes (cyclic extension), 72 °C for 10 min (final extension), and 4 °C. The final volume was 13 μL of each sample, being: 2μL of DNA (10 ng/μL), 1.50 μL of 10X Buffer (NH_4_SO_4_), 1.5 μL of MgCl_2_ (25 mM), 1.5 μL of dNTPs (2 mM), 1 μL of primer (R + F) (5 μM) and 0.12 μL of Taq-DNA polymerase (5 U/μL) (Invitrogen, Carlsbad, Califórnia, EUA). The amplification products were separated on 4% Metaphor agarose gel, stained with GelRedTM, and visualized through the MiniBis Pro photo-documentation system (Bio-Imaging Systems).

Two-hundred SSR primers were tested^16^. After screening, a set of 44 polymorphic primers was selected for the amplification reactions on the 96 plants previously sectioned.

### Statistical analysis

The genomic predictions was made using the following models: Bayesian Ridge regression (BRR—Bayesian Ridge regression), BayesA, BayesB, BayesB (setting the very low value of $\pi$, ${10}^{-5}$), BayesC and Bayesian Lasso (Bayesian Lasso—BL, assuming the marginal distribution as double exponential prior to the effects of markers). The general model for genomic predictions can be described in the matrix form as:

1 $$y=\mu +Xb+Wg+Zm+e$$

where: $y$ is the vector of the observations for each characteristic, $\mu$ is a vector of average, $b$ is the vector of blocks effects, assumed to be fixed, $g$ is the vector of family effects, assumed to be fixed, $m$ is the vector with the effects of the markers, assumed to be random, whose assumptions depend on the model used (described below), $W$ is the incidence matrix of the genotypes (coded as 0, 1, and 2 representing the allelic variations AA, Aa, and aa) of each plant and each marker and $e$ is the vector of the residues.

The models tested for the $W$ matrix assumptions were described in our provious work^17^, in summary:

Bayesian ridge regression (BRR)—is a Bayesian method in which it is assumed that all regression coefficients have common variance. Thus, for an additive model, all markers with the same allele frequency explain the same proportion of the additive variances, and have the same shrinkage effect^18^. For BRR it was assumed that:

$${a}_{i}\left|{\sigma }_{a}^{2}\sim N\left(0,{\sigma }_{a}^{2}\right);{\sigma }_{a}^{2}\right|{v}_{a},{S}_{a}\sim {X}^{-2}\left({v}_{a},{S}_{a}\right)$$

Bayes A—assumes that the markers with the same Minor allele frequency (MAF) to contribute differently to genetic variance, since the variances of the effect of the marks are heterogeneous^19^, Bayes A assumes:

$${a}_{i}\left|{\sigma }_{a\mathrm{i}}^{2}\sim N\left(0,{\sigma }_{a\mathrm{i}}^{2}\right);{\sigma }_{a\mathrm{i}}^{2}\right|gl,{S}_{a}\sim {X}^{-2}\left(gl,{S}_{a}\right);{S}_{a}|r,s \sim Gamma(r,s)$$

Bayes B—can be seen as a complement to Bayes A, since in addition to adjusting the markers with heterogeneous variances, Bayes B also assumes that some marks are not in LD with no gene, so they must have their effect zeroed, this mechanism of selection of marks is formulated through a mixture of distributions^19^, being the presuppositions of Bayes B given by:

$${a}_{i}|{\sigma }_{ai}^{2}\left\{\begin{array}{cc}\sim N(0,{\sigma }_{ai}^{2})& with \,probability \, 1-\pi \\ =0& with \,probability \,\pi \end{array}\right.$$

$$\pi |{\pi }_{0},p\sim beta({\pi }_{0},p)$$

$${\sigma }_{ai}^{2}|gl,{S}_{a}\sim {X}^{-2}(gl,{S}_{a})$$

$${S}_{a}|r,s\sim Gamma(r,s)$$

In the case of Bayes B2, π is not a parameter, but is fixed in such a way that the probability of a marker having zeroed effect is 10e − 5.

Bayesian Lasso (BL)—similarly to the philosophy of previous Bayesian methods, BL is assumed to have heterogeneous variances for the effect of marks, and BL also predicts that several marks are not in LD with no gene, however the selection of BL marks is indirectly through the marginal distribution of the marks effect, which is the double exponential (DE)^20^, a distribution more leptokurtic than the marginal prior distribution used in Bayes A and B that is a t Student^18^. The BL that will be adjusted in this study assumes:

$${a}_{i}|{\sigma }_{\varepsilon }^{2},{\tau }_{i}^{2}\sim N(0,{\sigma }_{\varepsilon }^{2}{\tau }_{i}^{2})$$

$${\tau }_{i}^{2}|\lambda \sim Exponencial(0.5{\lambda }^{2})$$

$$\lambda |r,s\sim Gamma(r,s)$$

According to^6,20^:

$${a}_{i}|\lambda \sim DE(\lambda )$$

Bayes Cπ—Habier et al. (2011) proposed the Bayes Cπ methodology, which is more parsimonious because it presents a common variance component between the effects of marks, so this method tends to present greater Bayesian learning, moreover, similar to Bayes B, Bayes Cπ also promotes the selection of marks that would not be in LD with any gene. The Bayes Cπ used in this study assumes:

$${a}_{i}|{\sigma }_{ai}^{2}\left\{\begin{array}{c}\sim N(0,{\sigma }_{a}^{2}) \,with \,probability \,1-\pi \\ =0 \,with \,probability \,\pi \end{array}\right.$$

$$\pi |{\pi }_{0},p\sim beta({\pi }_{0},p)$$

$${\sigma }_{a}^{2}|gl,{S}_{a}\sim {X}^{-2}(gl,{S}_{a})$$

The models were compared based on the Deviance Information Criterion (DIC) proposed by Spiegelhalter et al.^21^. The DIC can be described as follows $DIC=D(\acute{\theta })+2{p}_{D},$ in which the first term is a Bayesian model adjustment measure ($D(\acute{\theta }))$, which is defined as the a posteriori mean of deviance and the second component (${p}_{D}$) measures the complexity of the model through the effective number of parameters. Posterior probabilities of the models were calculated using the approximation presented by Wilberg and Bence^22^ to facilitate the interpretation of DIC values in terms of the superiority of one model over the other, in which it is given by:

$$p({M}_{t}\vee l)=\frac{\mathrm{e}\mathrm{x}\mathrm{p}(-{\Delta }_{t}/2)}{\sum _{t=1}^{6}\mathrm{e}\mathrm{x}\mathrm{p}(-{\Delta }_{t}/2)},t=\mathrm{1,2},\mathrm{3,4},\mathrm{5,6}$$

where: $p({M}_{t}\vee l)$ is the a posteriori probability of model $t$, ${\Delta }_{t}$ is the difference between the DIC of model $t$ and the model with the lowest DIC.

A cross-validation method was used to access the model with best fit.For each model in each trait, the data were splitted into two subsets. The first one was composed by randomly 75% and was used to estimate the marker effects. The second one, the validation partition was 25%, had their phenotypes predicted by the marker effects estimated in the training set. The process was repeated 8 times (folds), each time estimating the correlation between predicted and observed phenotypic data and predicted accuracy (ratio between fold correlation and square root of heritability)^23^.

We also estimate the additive genetic variance using the marker variance ${\sigma }_{a}^{2}=\sum 2pq\text{var}(mar\mathrm{k}\mathrm{e}\mathrm{r})$, and heritability based on estimates of additive genetic variances and residual variances ${\mathrm{H}}^{2}={\sigma }_{a}^{2}/({\sigma }_{a}^{2}+{\sigma }_{e}^{2})$, complementarily, we estimate the genetic correlations based on the genetic values predicted for the evaluated characteristics.

A complete description of the calculation of heritability and the specifications of the probability distributions of the general model effects above, for the use of Bayesian methods, can be found in Pérez and de Los Campos^19^. All Bayesian analyzes were performed in the BGLR package^19^ of the R software^24^, with the BGLR function adjusted for 1E6 iterations with the first 2E5 cycles discarded as burn-in and thin assuming the value 4. Plants (individuals) were ranked using the model that shows the best fit, according to genomic genetic value, given by ${\widehat{y}}_{j}=\sum _{i}{Z}_{i}{\widehat{m}}_{i}$.

## Results

Six Bayesian models were applied to detect the effect of the markers along with phenotypic data from a guava population. In the modeling process, cross-validation with eight folds was used to obtain some adjust parameters of the models in all folds (Table 1). Among the models used, the Bayesian Ridge Regression model—BRR presented the lowest mean value considering a comparative adjustment value (< *DIC—Deviance Information Criterion*) according to the parameters used in the trait soluble solids content (°BRIX).

**Table 1:** Adjustment quality of six Bayesian models: BL, BRR, BayesA, BayesC, BayesB, and BayesB with π = 1e − 5 (BayesB2) to associate SSR markers and phenotypic data in *P. guajava* in the traits of soluble solids content, fruit mass, pulp mass, number of fruits per plant and production per plant.

	DIC	∆	Wprob	ER	r	*p* value
	**Soluble solids content**					
BRR	1348.95	0.00E + 00	5.68E − 01	1.00E + 00	0.65	1.76E − 11
BayesA	1352.36	3.41E + 00	1.03E − 01	5.50E + 00	0.65	1.82E − 11
BayesL	1455.71	1.07E + 02	3.73E − 24	1.52E + 23	0.65	3.34E − 11
BayesC	1352.66	3.71E + 00	8.90E − 02	6.38E + 00	0.65	2.50E − 11
BayesB	1353.17	4.22E + 00	6.87E − 02	8.26E + 00	0.65	3.29E − 11
BayesB2	1351.35	2.40E + 00	1.71E − 01	3.31E + 00	0.35	3.00E − 10
	**Fruit mass**					
BRR	4330.52	7.03E − 01	2.58E − 01	1.42E + 00	0.65	5.53E − 12
BayesA	4332.11	2.29E + 00	1.16E − 01	3.15E + 00	0.65	8.88E − 12
BayesL	4377.44	4.76E + 01	1.67E − 11	2.20E + 10	0.64	1.20E − 11
BayesC	4331.05	1.23E + 00	1.98E − 01	1.85E + 00	0.65	7.59E − 12
BayesB	4333.38	3.57E + 00	6.16E − 02	5.95E + 00	0.65	1.04E − 11
BayesB2	4329.82	0.00E + 00	3.66E − 01	1.00E + 00	0.52	2.46E − 12
	**Pulp mass**					
BRR	4179.22	8.37E − 01	2.64E − 01	1.52E + 00	0.64	3.04E − 11
BayesA	4181.10	2.71E + 00	1.03E − 01	3.89E + 00	0.64	5.58E − 11
BayesL	4224.53	4.61E + 01	3.83E − 11	1.05E + 10	0.62	7.56E − 11
BayesC	4180.16	1.78E + 00	1.65E − 01	2.43E + 00	0.63	4.36E − 11
BayesB	4181.99	3.61E + 00	6.60E − 02	6.08E + 00	0.63	6.04E − 11
BayesB2	4178.39	0.00E + 00	4.01E − 01	1.00E + 00	0.50	1.28E − 11
	**Number of fruits**					
BRR	980.19	6.57E − 01	1.71E − 01	1.39E + 00	0.82	4.36E − 13
BayesA	981.18	9.87E − 01	1.45E − 01	1.64E + 00	0.79	8.92E − 13
BayesL	980.75	5.58E − 01	1.79E − 01	1.32E + 00	0.76	4.31E − 13
BayesC	981.36	1.17E + 00	1.32E − 01	1.79E + 00	0.80	2.88E − 13
BayesB	980.15	0.00E + 00	2.37E − 01	1.00E + 00	0.79	2.39E − 13
BayesB2	981.31	1.12E + 00	1.35E − 01	1.75E + 00	0.73	8.51E − 13
	**Production per plant**					
BRR	1825.73	8.71E − 01	1.99E − 01	1.55E + 00	0.84	2.02E − 13
BayesA	1826.51	1.64E + 00	1.36E − 01	2.27E + 00	0.82	5.34E − 13
BayesL	1828.81	3.95E + 00	4.28E − 02	7.20E + 00	0.77	4.08E − 13
BayesC	1825.92	1.06E + 00	1.81E − 01	1.70E + 00	0.82	1.47E − 13
BayesB	1826.55	1.69E + 00	1.33E − 01	2.33E + 00	0.81	3.53E − 13
BayesB2	1824.86	0.00E + 00	3.08E − 01	1.00E + 00	0.74	1.10E − 12

The bias values were obtained by eight-fold cross-validation (88% of the data for training and 12% for validation), in the same sample sets for each model.

DIC = deviance information criterion; Del (delta) = difference between the highest and the lowest DIC value; Wprob = posterior probability model; ER = evidence ratio; Error = error attributed to Wprob; r = correlation between predicted by the model and reserved validation data; *p* value = significance of the correlation.

**Figure 1:**
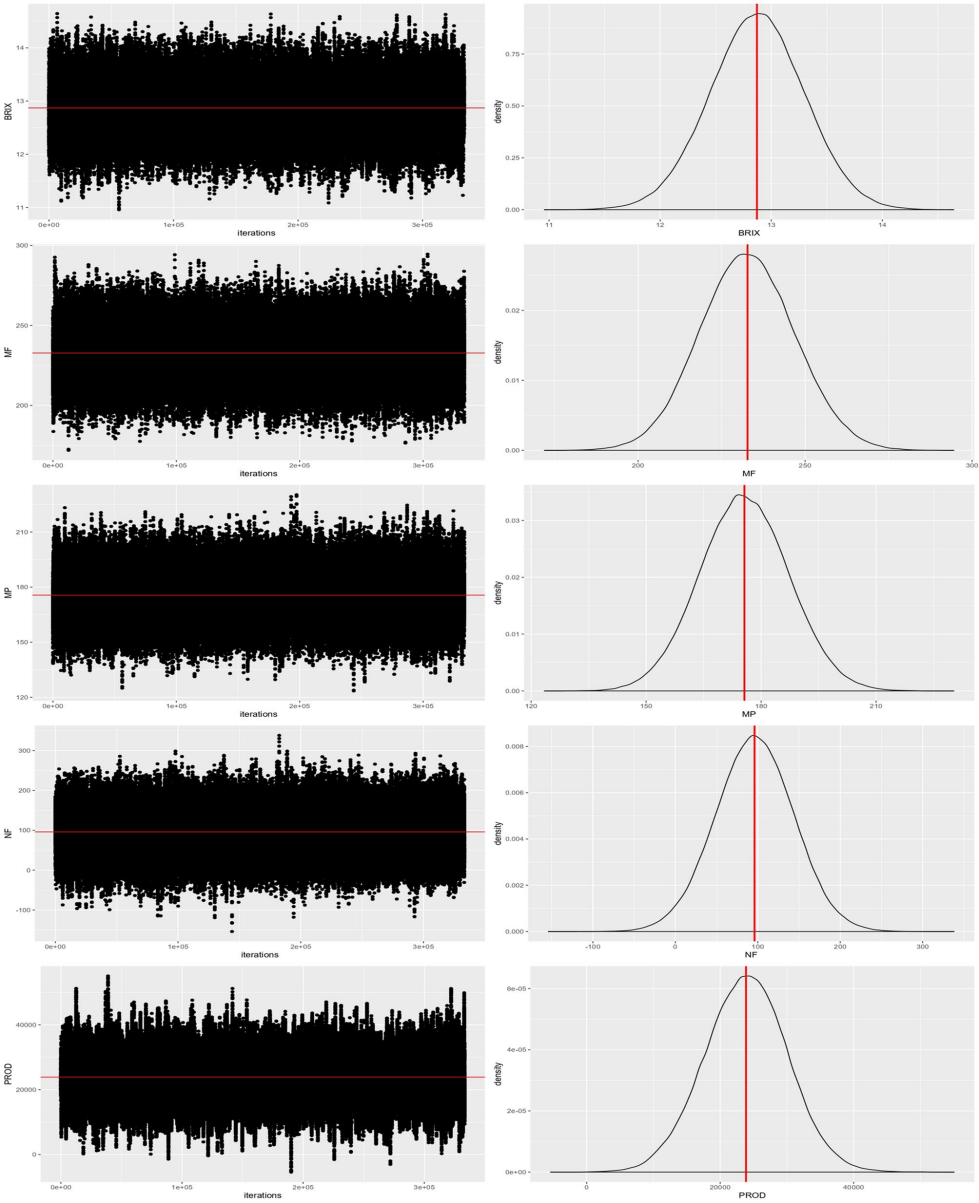
Markov and Monte Carlo chains with mean values (red line) and distribution curve for five traits observed in guava, generated to relate SSR marks to phenotypic observations.

The DIC is particularly useful in problems of Bayesian selection models, where the posterior distributions of the models were obtained by the Markov Chain Monte Carlo simulation (MCMC). DIC is an asymptotic approach as the sample size becomes large, like the AIC. It is only valid when the posterior distribution is approximately normal multivariate. Thus, the chain convergences and the posterior distribution (normal distribution) were verified for all traits in the BRR model (Fig. 1).

A good stabilization of the Markov and Monte Carlo chains was observed with the mean values corresponding to observed phenotypic means. The posterior density curves of the chains showed normal distribution in all traits. Therefore, it is possible to use DIC values to select the models safely.

Deviations (∆) of information criteria were also obtained for each trait concerning the lowest value, assumed as the model that presented the best fit to the data. From these parameters, auxiliaries were also obtained in the classification of models as values of posterior adjustment probability of the model (Wprob) and the evidence ratio (ER) for the models. All adjust parameters of the BRR model were superior to the other Bayesian models used for the SSC trait.

Besides the adjustment values, for the model choice, we consider the model’s ability to predict the phenotypic values of a sub-sample with random individuals, in each fold of the cross-validation. The mean values of the predicted correlation and the observed phenotypes (r), together with a probability value of r, had no linear correlation (Table 1).

For the SSC trait, the BRR model also showed the highest r value with the lowest probability, being a consistent correlation between the subsamples. The other models performed very similarly, except for the BayesL model, where a discrepant DIC value was observed, and the BayesB2 model, which despite showing a good fit with a similar DIC, presented a low predictive capacity with r = 0.35 concerning BRR with r = 0.65.

A similar result in the model’s adjustment and prediction criteria was observed for the other traits, such as the number of fruits per plant (NF). In NF, adjusted values of the very similar models close to 980.19 (DIC) were observed, with the BRR model chosen by the best predictive capacity with r = 0.82 for 0.65 for FM, 0.64 for PM, and 0.84 for PROD.

With the model adjustment criteria very close between the models used and great differences between the predictive power of each model within the traits, it was possible to observe that choosing a value of π for the BayesB model caused an overfit of the model. It was observed that the predictive power of the BayesB model, in most cases, presented the worst results (r).

It is worth mentioning that the Bayesian models take considerable time to be executed. Even with the advancement of computational power, the resolution of more complex models requires a longer processing period. This is widely known information, but little measured, which must be considered when choosing the model. In this study, the time invested in solving the Bayesian models was measured by repeating each chain ten times in a loop (Table 2).

**Table 2:** Estimates of time averages for solving different Bayesian models with 10^6^ iterations, burn-in of 10^4^, and chain sampling equal to 3.

Model	Processing time*
Bayesian Ridge Regression—BRR	15.3 h (+ − 0.15)
BayesA	15.35 h (+ − 0.10)
BayesB	15.32 h (+ − 0.13)
BayesB $\pi = {10}^{-5}$	15.3 h (+ − 0.10)
BayesC	15.5 h (+ − 0.2)
Lasso Bayesian	14.95 h (+ − 0.5)

*A 2.7 GHz Intel I7-7500U processor core was used. The calculations were performed with the BGLR package^19^ (version 1.0.8) in R language^24^ (version 3.5.1).

Narrow-sense heritability values were estimated for the traits observed in guava with the model that showed the best performance (Table 3). The extremes of the values were for the soluble solids content (TSS = 0.32) with the highest observed heritability value, and the number of fruits per plant (NF = 0.07) had the lowest heritability value. In general, heritability values were low but accompanied by deviation and accuracy measures; they can provide more accurate estimates for the advancement of generations in the breeding program. The values of the heritability deviation measure were low. This indicates more precise values for heritability, as opposed to estimates of heritability obtained punctually, as is commonly done. Error estimates of heritability were obtained with the estimate of heritability in each iteration of cross-validation, thus being estimated in several subsets that represent the population.

**Table 3:** Predict accuracy and standard deviation of heritability for soluble solids content (TSS), fruit mass (FM), pulp mass (PM), number of fruits per plant (NF) and production per plant (PROD) observed in guava (*Psidium guajava*), estimated using a model with SSR markers and Bayesian ridge regression—BRR.

	H2	Standard deviation	Predict accuracy
SSC	0.3261	0.0706	0.6302
FM	0.1581	0.0215	0.8779
PM	0.1478	0.0310	0.9708
NF	0.0732	0.0146	0.6939
PROD	0.1058	0.0154	0.5095

In predictive accuracy, high values were observed, which is a good indication that the estimated heritability represents the population very well. In particular, the predictive accuracy value for the PM trait, estimated at 0.9708. However, when observing the predictive accuracy of heritability of PROD, the trait of main interest, a value close to 0.51, was obtained, which is low. Very similar results were observed for fruit mass and pulp mass. The heritability values were 0.1581 and 0.1478 from FM and PM, respectively. The standard deviation of heritability was also close and low. Only the predictive accuracy was better in PM than FM, indicating that the volume inside the fruits depends less on the size of the fruit, being more random or influenced by another factor not observed in this experiment.

**Figure 2:**
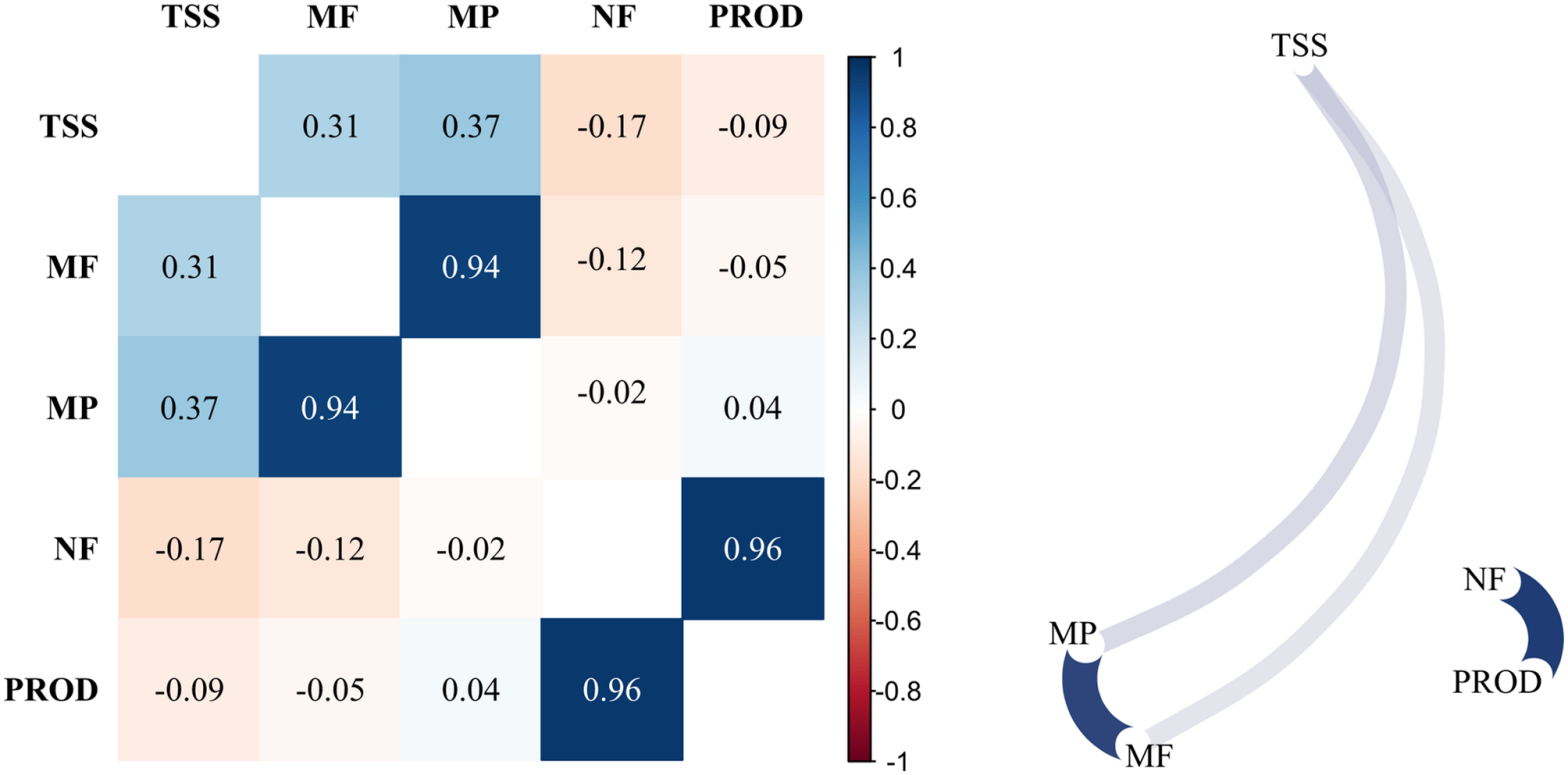
Genetic correlation between the soluble solids content (TSS), fruit mass (FM), pulp mass (PM), number of fruits per plant (NF), and production per plant (PROD) observed in guava (*Psidium guajava*), estimated using a model with SSR markers and Bayesian ridge regression—BRR.

With the matrix of the individuals’ marks and the weight that each marker received in the Bayesian ridge regression model, the individuals’ genetic values of the traits were estimated, and the genetic correlation matrix between the traits was obtained (Fig. 2). A high linear correlation was observed between PM and FM (0.9393) and between NF and PROD (0.9641). A correlation was also observed between soluble solids content and two traits of the fruit, with a value of 0.3060 between SSC and FM, and 0.3705 between SSC and PM. It was also observed that SSC showed a negative correlation with PROD and NF, but there were no significant correlations.

The individuals contained in Table 4 were selected because they present positive values in all traits. However, it is possible to use a selection index if the objective is to select new individuals to compose a new population within a breeding program. Families 8, 10, and 17 were the families that contained more individuals in ordering the genetic values considering the production per plant. Thus, these families have low variability among themselves, but with a high productive capacity, being recommended for selection and continuity in trials of Value for Cultivation and Use.

**Table 4:** List of selected individuals who presented positive genetic values in the traits soluble solids content (TSS), fruit mass (FM), pulp mass (PM), number of fruits per plant (NF), and production per plant (PROD).

Individual	TSS	FM	PM	NF	PROD
B1F15P12	0.76	10.68	8.45	24.57	4507.96
B2F8P4	0.19	6.54	4.12	13.11	3115.68
B2F1P4	0.64	4.99	12.08	14.53	2958.55
B1F15P10	2.51	18.14	16.13	1.25	2848.35
B1F2P8	1.21	33.01	29.67	13.28	2268.82
B2F2P10	0.86	12.18	12.33	16.93	2206.82
B2F3P11	0.89	19.74	19.79	9.29	1421.55
B1F8P1	1.04	18.97	15.93	7.67	1264.91
B2F12P9	0.96	6.90	5.19	9.00	1245.41
B2F17P2	0.27	7.61	4.86	6.15	622.86

The individuals were classified in descending order considering the PROD.

## Discussion

From a Bayesian approach, the effects of markers can be estimated together to predict the genomic values for a quantitative trait without performing the marker selection. This approach is called genomic selection. Several penalized and of estimation methods of Bayesian contraction are available, for example, Bayesian counterparts of Ridge Regression (Ridge Regression—RR)^25^, Least Absolute Shrinkage and Selection Operator (Least Absolute Shrinkage and Selection Operator—LASSO)^26^, as well as models such as BayesA and BayesB and their extensions^9^. These models are frequently tested for different crops of interest; however, for guava, this information is still scarce. In this study, the performance of six Bayesian models for adjusting SSR markers in guava is discussed and estimated parameters of interest to the breeder in a breeding program.

Although there are differences between the methods, in a priori assumptions about the effects of the markers, it was observed that adjustment parameters of the models were similar. No evident difference was detected for any of the traits, mainly for DIC. Thus, the models were chosen, considering not only the adjustment parameters but also their predictive capacity and how they behave concerning the markers to generate the regressive model.

BayesL produces a stronger shrinkage of regression coefficients close to zero and less shrinkage for those with large absolute values, leading to a scarcer model. By other hand, BRR reduces strongely regression coefficients that have large absolute values^27^. Thus, it was observed that BayesL presented a median performance, possibly because the number of significant marks, with great effects on the model was too scarce to explain the quantitative traits evaluated. Intuitively the reverse occurred with the BRR model, which considered the effects of marks more, generating a model with more marks to explain traits controlled by several genes. This means that the distribution of the marks was, on average, slightly less than peaks for the effects research grid in the BRR model.

Studies that seek the best models for different species are important to direct breeding programs. For example, for another perennial plant (*Passiflora edulis*), it was observed that the BayesC model was the best model for several traits evaluated in this species^17^. This model assumes a common variance for all effects of markers but also assumes that some markers do not affect^28^. Thus, genes with the same allelic frequency probably explain the same portion of genetic variation, suggesting that several genes with few effects control the traits, as the quantitative traits observed in this study. In the results, it was possible to observe that this model also presented a satisfactory performance for traits in guava, being able to be chosen as an alternative model.

Similar results between Bayesian methods such as BayesA and BayesB and other derivatives of these were also observed^28^, as obtained in this work. This similar result was already expected since the models have few variations between them. For example, BayesB and BayesA are more tolerant of the assumption of common variance between the effects of the markers. A priori assumed in these models for the effect of a jth marker is a joint distribution with a probability π for the beta for the mark equal to zero.

When the BayesB model was proposed, π was suggested with a value close to 0.95^9^. However, with a few marks, it is possible to choose lower values for π, where BayesB with π reduced to zero is equivalent to the BayesA model. As possible, overfitting of the BayesB model was observed when we used a value of π = 1e − 5; it was forced that the marks had a high probability of influencing the trait of interest. Thus, a model was obtained in which the betas referring to the brands fitted very well to explain the sub-sample in each fold of the cross-validation, but failed to predict the validation sample as observed for most traits (Table 1).

If only the model’s adjustment parameters such as the DIC, which are widely used, had been used, perhaps it would not be observed that the predictive power of the BayesB model had the worst performance. This highlights the importance of cross-validation. Cross-validation was used to assess how the results of one statistical model resemble another set of data. For example, how an adjusted model will predict data that was not used to adjust the model. Predicting the performance of genotypes with phenotypes yet to be observed (for example, newly developed lines or lines that have been evaluated in a few environments) is essential in plant breeding. Therefore, cross-validation appears to be a natural way to assess model performance from the breeder’s perspective^29^.

Simulation studies have shown that genomic selection using markers alone can adjust the model to an accuracy of up to 85%^9^. The accuracy of 85% is the correlation between the true genetic values and the predicted values of individuals in the next generation. True genetic values are known only in simulation studies. In the analysis of real data, the predictability of a model must be extracted from a cross-validation study. The predictability obtained from cross-validation and the quality of the model’s fit do not necessarily agree with each other. Starting with a small number of markers, both can increase as the number of markers increases. Further increasing the number of markers may continue to increase the quality of the model’s fit, but predictability may drop^30^.

The heritability coefficient influences the prediction of genomic genetic values, predictive capacity, and association analysis across the genome. With greater heritability of phenotype, there are improvements in the identification of individuals to be used as parents in the next generations, also favoring the identification of regions associated with a characteristic of interest^17^.

The heritability of the TSS characteristic was the highest observed, and the value corroborates within the range with a study that evaluated a large population of guava trees in India^12^. The authors also detect a correlation between this trait and fruit mass, allowing an indirect selection. It is also suggested that there may be a possible non-additive effect on the genes controlling this characteristic, as they observed a phenotypic variance greater than the genotypic variance. Our model showed low predict accuracy for heritability despite the higher value. Also, approximately 40% of the subsample of validation, the model presented a biased prediction, also corroborating the idea of gene action with non-additive effects from this characteristic.

For the other traits, the heritability values were low, as expected. The values generally reported for traits such as fruit mass, pulp mass, number of fruits, and production are generally close to 0.60^31^. Our estimates are possibly lower because they are estimates from a model that considers the effect of marks, and the usual estimates are obtained from phenotypic data that have many more sources of variation, often not considered. Despite being low, heritability showed good predict accuracy in cross-validation, reaching 0.97 for the pulp mass.

Pulp mass is strongly correlated with the fruit mass, which from the point of view of plant physiology was already expected. FM and PM are traits obtained in similar ways, where one measures the mass of the whole fruit, and in the other, the placenta containing the seeds is removed, a part that does not matter in the processing of the fruit. Both traits showed similar heritability values of 0.14 and 0.15, which were superior to the traits of interest regarding production (NF and PROD). Generally, collinearity is observed between these two traits, and this collinearity is particularly interesting for studies of correlations between traits in guava, which may involve modeling structural equations such as path analysis, which seek traits that can be selected indirectly.

As the heritability is very similar in the two traits, and the genetic correlation between them is also high, a program can direct the selection of individuals with higher pulp mass with the indirectly selecting based on fruit mass. In the selection stages, there is a big difference in time and resource spent between just measuring the mass of a set of fruits versus opening a fruit removing the placenta, and measuring the mass of the pulp.

In the traits number of fruits and production, heritability was very low, together with estimates of predict accuracy. Since these are also quantitative traits, usually controlled by many genes, low heritability was expected. However, despite predicting the validation subsample with more than 50%, probably our model was not able to capture all the effects for these traits since the model has an unsatisfactory performance.

It is worth mentioning that these traits evaluated are highly influenced by the environment, and especially by management^32^. For example, a common crop handling in guava trees is the pruning and a subsequent thinning of new shoots that arise after pruning. This serves to control both the plant height to facilitate harvesting and the number and size of fruits. Thus, the inflorescences that originated the fruits appear in buds in the axils of the new shoots. If many shoots are maintained after pruning, the number of fruits tends to increase, but the fruit mass is less due to the greater distribution of the available resources of the parent plant. This leads us to look for a correlation between the number of fruits and production with, for example, the mass of the fruit, which was not found here, or at least it is a non-linear correlation since the correlations between NF and FM are close to zero (Fig. 2).

Different genetic values were observed among the selected individuals; a possible explanation for this fact is that the population has high genetic variability. This implies in the differences between the genetic values of individuals, making them more pronounced, making it easier for the methods to classify individuals with greater accuracy.

## Conclusion

The Bayesian ridge regression model showed the best results and was chosen to predict the genetic values of individuals in the traits soluble solids, fruit mass, pulp mass, number of fruits, and production per plant. Heritability values showed good predict accuracy. Genetic correlations were obtained to verify the relationship between traits, and the model showed strong correlations between some traits, allowing the indirect selection.

## Data Availability

The full phenotypic information, breeding values, scripts and chains generated used in this study, have been submitted at the *Open Science Framework* and was awarded the public doi identifier: https://doi.org/10.17605/OSF.IO/T8X7U.
